# FUTURE OF CAREGIVING: INNOVATIVE SOLUTIONS AND CHALLENGES WITH NOVEL NONPHARMACOLOGICAL INTERVENTIONS

**DOI:** 10.1093/geroni/igae098.1776

**Published:** 2024-12-31

**Authors:** Jyoti Savla, Lauren Hagemann

**Affiliations:** Chair; Co-Chair

## Abstract

As we explore the evolving landscape of family caregiving, it becomes clear that caregiving roles offer fulfillment but also pose unique mental health challenges for caregivers of persons living with dementia. These challenges stem from expanded responsibilities and managing dementia-related behaviors. Research emphasizes the intertwined well-being of caregivers and their care recipients, highlighting the need for interventions that support caregivers’ mental health and improve their capacity to address their care recipients’ needs. This symposium aims to assess the effectiveness of novel non-pharmacological interventions, marking a step forward in our approach to caregiver support and improving caregiving dynamics overall. Martinsdale-Adams et al. explore the Annie Caregiver Text program, a text-message intervention based on the REACH-VA protocol, showing its success in improving the management of dementia-related behaviors and reducing caregiver stress. Lee et al. examine the Powerful Tools for Caregivers (PTC) program, assessing its effectiveness in enhancing self-care and communication skills, whether delivered in-person or remotely. Savla et al. share results from an RCT that compares the effectiveness of a mindfulness-based intervention, Practice of Acceptance, Awareness, and Compassion in Caregiving (PAACC), with REACH-VA, focusing on reducing caregiver anxiety and increasing compassion. Ruggiano et al. analyze the Alabama Caregiver Connect initiative, aimed at simplifying access for caregivers to essential dementia care information through interactive, AI-enhanced web tools, including ChatGPT and chatbots. Together, these empirically-validated programs offer novel non-pharmacological options for managing caregiving responsibilities and addressing dementia-related behaviors of relatives, exploring the challenges and solutions for implementing these programs for family caregivers.

